# Lipoprotein size is a main determinant for the rate of hydrolysis by exogenous LPL in human plasma

**DOI:** 10.1016/j.jlr.2021.100144

**Published:** 2021-10-26

**Authors:** Oleg Kovrov, Fredrik Landfors, Valeria Saar-Kovrov, Ulf Näslund, Gunilla Olivecrona

**Affiliations:** 1 Department of Medical Biosciences, Umeå University, Umeå, Sweden; 2 Department of Public Health and Clinical Medicine, Umeå University, Umeå, Sweden; 3 Department of Pathology, CARIM School for Cardiovascular Diseases MUMC+, Maastricht University, Maastricht, The Netherlands; 4 Heart Centre and Department of Public Health and Clinical Medicine, Umeå University, Umeå, Sweden

**Keywords:** plasma triglyceride metabolism, apolipoproteins, angiopoietin-like proteins, lipidomics, isothermal titration calorimetry, VLDL particle size, exogenous LPL, lipid signature, capillaries, ANGPTL, angiopoietin-like protein, Apo, apolipoprotein, DOC, deoxycholate, GPIHBP1, glycosylphosphatidylinositol-anchored HDL-binding protein 1, HSPG, heparan sulphate proteoglycan, ITC, isothermal titration calorimetry, TG, triglyceride, TRL, triglyceride-rich lipoprotein, VIPVIZA, visualization of asymptomatic atherosclerotic disease for optimum cardiovascular prevention, WAT, white adipose tissue

## Abstract

LPL is a key player in plasma triglyceride metabolism. Consequently, LPL is regulated by several proteins during synthesis, folding, secretion, and transport to its site of action at the luminal side of capillaries, as well as during the catalytic reaction. Some proteins are well known, whereas others have been identified but are still not fully understood. We set out to study the effects of the natural variations in the plasma levels of all known LPL regulators on the activity of purified LPL added to samples of fasted plasma taken from 117 individuals. The enzymatic activity was measured at 25°C using isothermal titration calorimetry. This method allows quantification of the ability of an added fixed amount of exogenous LPL to hydrolyze triglyceride-rich lipoproteins in plasma samples by measuring the heat produced. Our results indicate that, under the conditions used, the normal variation in the endogenous levels of apolipoprotein C1, C2, and C3 or the levels of angiopoietin-like proteins 3, 4, and 8 in the fasted plasma samples had no significant effect on the recorded activity of the added LPL. Instead, the key determinant for the LPL activity was a lipid signature strongly correlated to the average size of the VLDL particles. The signature involved not only several lipoprotein and plasma lipid parameters but also apolipoprotein A5 levels. While the measurements cannot fully represent the action of LPL when attached to the capillary wall, our study provides knowledge on the interindividual variation of LPL lipolysis rates in human plasma.

CVD is one of the main causes of death and morbidity in modern society (1). Recent genetic evidence suggests that plasma triglycerides (TGs), triglyceride-rich lipoproteins (TRLs), and TRL remnants play major roles in the progression of CVD (2). TG levels in the blood are to a large extent regulated by the action of LPL (3). Thus, there is currently a great interest in LPL and its regulators.

LPL (Enzyme Commission number: 3.1.1.34) hydrolyses TG that are transported in the core of chylomicrons and VLDL into FFAs and monoglycerides. The lipolysis products are taken up by many tissues: by white adipose tissue (WAT) for storage, by brown adipose tissue for heat production, or by muscle tissues for energy production (3, 4). The synthesis rate of LPL is found to be relatively constant because of rather stable mRNA levels (3, 5). The turnover of the enzyme is too slow to respond to the metabolic cues that are repeated several times during the day after food ingestion or bouts of physical activity (3, 4, 5, 6, 7). Therefore, a major part of the LPL regulation occurs post-translationally: either in the parenchymal cells of muscle and adipose tissue; during the secretion and transendothelial transport of the enzyme by the glycosylphosphatidylinositol-anchored HDL-binding protein 1 (GPIHBP1) to the site of action on the capillary endothelium; or, during the action of LPL on the TRL. Other important control proteins for LPL include the apolipoproteins (Apos) that are found on the surface of TRL and angiopoietin-like proteins (ANGPTLs) that regulate the amount of active LPL in the tissues in response to metabolic and hormonal signals (8).

The exchangeable Apos are relatively small amphipathic proteins that are found both on TRL and on HDL. When the TRLs are depleted of their TG, the exchangeable Apos leave the shrinking particles and relocate to HDL, which serve as a reservoir for Apos in the blood (9, 10). HDL-bound Apos can then be recycled into newly synthesized lipoproteins. Four of the exchangeable Apos were previously shown to regulate LPL activity—ApoC1, ApoC2, ApoC3, and ApoA5. ApoC2 serves as a cofactor for LPL—it helps LPL to stay attached to the TRL particle until it gets sufficiently lipolyzed, but the exact mechanism of action for ApoC2 on LPL is not known (11). ApoC1 and ApoC3, on the other hand, are inhibitors of LPL activity. Their mechanism of action is still under debate. It was proposed that they inhibit TG hydrolysis either by making the TG from the core of TRL inaccessible to LPL or by displacing ApoC2 from the TRL (12, 13). Studies on ApoC3 demonstrate that LPL-independent inhibition of receptor-mediated uptake of lipoproteins and remnants may be more important than previously thought (14). ApoA5 is a more recently identified activator of LPL. The mode of action of ApoA5 is still an enigma—it is present in minute amounts in plasma compared with the other Apos, and the effect of ApoA5 on LPL activity has been difficult to replicate in vitro. In previous studies, ApoA5 was shown to bind to heparan sulphate proteoglycans (HSPGs) and to GPIHBP1 (15, 16). Thus, ApoA5 may serve as a bridge between TRL and the capillary wall and thereby bring TRL closer to LPL.

The ANGPTL family consists of eight proteins, three of which are important in TG metabolism through their action on LPL. ANGPTL3, ANGPTL4, and ANGPTL8 act as irreversible inactivators of LPL (8, 17, 18). ANGPTL4 is the most studied protein of the three in relation to its action on LPL. ANGPTL4 is produced at various sites in the body but was proposed to act on LPL predominantly in WAT. During fasting, ANGPTL4 levels in WAT increase, LPL becomes inactivated, and the TG in VLDL are directed to energy-demanding tissues like skeletal muscle and heart (19, 20). During exercise, the ANGPTL4 levels in resting muscles increase, thus directing plasma TG to contracting muscles (21). ANGPTL3 is mostly produced in the liver and inactivates LPL in metabolically active tissues (22). ANGPTL3 works together with ANGPTL8, which has no effect on its own, but is upregulated in the fed state and greatly enhances the negative effect of ANGPTL3 on LPL activity (17, 18, 23, 24). ANGPTL8 may also form complexes with ANGPTL4 that, in contrast to the ANGPTL3/8 complexes, have reduced effect on LPL activity compared with that of ANGPTL4 alone (17, 18).

Apos and ANGPTLs are the main protein regulators of LPL activity, but the enzyme also responds to other factors. Effects of TRL size, lipid composition, and other biochemical and biophysical characteristics of the substrate lipoproteins on the activity of LPL were previously reported (25, 26). However, in most studies, these factors are ignored. The aim of the present study was to apply the novel isothermal titration calorimetry (ITC)-based method to measure the activity of LPL on TRL directly in human plasma (27). ITC was described as an alternative method to currently available activity assays for LPL. It allows the use of intact lipoproteins, without need for their isolation from plasma, and therefore preserving the natural equilibrium between the surface components of the lipoproteins and other plasma factors. In comparison with other methods to measure LPL activity, ITC is not affected by the high background levels of FFA in plasma, and, being label free, ITC does not suffer from pan-assay interference compounds. ITC is currently the only technique that allows comparisons of plasma samples from different individuals with regard to their ability to support LPL-mediated TG hydrolysis. Here, we have studied lipolysis of plasma samples from a randomized subgroup of middle-aged participants of the visualization of asymptomatic atherosclerotic disease for optimum cardiovascular prevention (VIPVIZA) study (28, 29).

## Materials and methods

### VIPVIZA trial

Human blood samples were obtained from a randomly selected group from the VIPVIZA cohort at their 3-year follow-up visit. VIPVIZA is a randomized controlled trial with the aim to improve the diagnosis and prevention of CVD (28, 29). VIPVIZA subjects were recruited from the Västerbotten Intervention Program, which is a population-based study integrated into the primary health care services. It includes the assessment of traditional CVD risk factors as well as health counseling for 40-, 50-, and 60-year-old individuals (30). About 3,532 participants from the Västerbotten Intervention Program, with at least one CVD risk factor, were enrolled into the VIPVIZA study. Out of the 3,532 individuals, 118 participants were randomly selected for the in-depth analysis of their plasma. One individual was excluded because of a broken tube. The study was performed in accordance with the ethical principles set forth in the Declaration of Helsinki and was approved by the Regional Ethics Review Board of Umeå (diary no.: 2016-245-32M).

### Sample preparation

Blood was taken from overnight fasted study participants into EDTA-containing tubes by trained and qualified medical professionals. The tubes were centrifuged for 15 min at 2,000 *g* at room temperature. Plasma was recovered and immediately aliquoted into small volumes that were stored at −80°C. ITC experiments and analyses by NMR were performed on plasma that was thawed only once. The ELISA experiments were performed with the aim to keep repetitive freezing and thawing of the plasma sample to a minimum. At worst, samples were frozen and thawed three times before performing an ELISA experiment. The amount of freeze and thaw cycles was consistent throughout the ELISA assays, meaning that all samples used in the specific assay were frozen and thawed for equal number of times.

The plasma samples for the ITC experiments were prepared as described previously (26). In brief, immediately before the experiments, 360 μl of plasma was mixed with 40 μl of 200 mM Tris buffer (pH 7.4) to a final concentration of 20 mM Tris-HCl. The LPL used in the experiments was purified from bovine milk, as described previously (31). A stock solution of LPL was diluted to a concentration of 40 nM in ice-cold 10 mM Tris (pH 8.5) containing 4 mM sodium deoxycholate (DOC). This solution was aliquoted in small volumes and stored at −80°C. DOC stabilizes LPL and protects it from both temperature- and ANGPTL-induced inactivation (32, 33). Dilution of DOC in solutions containing albumin abolishes this protective effect because of the high-affinity binding of DOC to albumin (27, 34).

Samples for NMR and ELISA experiments were treated in accordance with the protocols from the service providers and manufacturers, respectively.

### ITC

LPL activity in plasma samples was measured using an assay based on ITC, which was described previously (27). Experiments were performed using a MicroCal Auto-iTC200 (GE Healthcare) instrument. Only 20 samples were loaded per run in order to ensure the stability of both LPL and plasma during the experiment. To adjust for interassay (12.5%) and intra-assay (5.8%) variations, two standards were run before and after the samples. The plate loaded with the individual plasma samples was stored at 4°C with 400 μl of buffered human plasma per well or with 200 μl of DOC-stabilized LPL per well. The ITC sample cell was equilibrated with 20 mM Tris buffer (pH 7.4) before each experiment. Measurements of enzyme activity were performed at 25°C with 200 μl of buffered plasma in the ITC sample cell and 40 μl of DOC-stabilized LPL in the syringe. The temperature was selected because of the fact that the assay was validated at 25°C temperature (27). The stirring speed in the sample cell was 600 rpm. The injections of LPL were started after a delay of 60 s to provide an initial baseline. In order to remove gas bubbles from the syringe, the first injection was 0.2 μl and lasted for 100 s. This was used as the zero point in later calculations. Subsequently, three more injections of 5 μl were made. Each injection increased the LPL concentration by 1 nM. The enzyme was allowed to act on the plasma lipids for 300 s, ensuring that there was a linear response to the increased concentration of LPL under the experimental conditions. After each experiment, the MicroCal Auto-iTC200 system was washed with MilliQ water, followed by 10% Decon90 (Decon Laboratories Ltd), and finally with 100% methanol. Data were analyzed in Microsoft Excel, and the rate of heat production was expressed as microjoule per second. Peaks in heat production occurred because of protein–protein/protein–lipid interactions and the effects of mixing. These peaks were omitted from the analyses. The difference in the baselines was calculated as was done in the previous study (27) and exported into R software (free from The R Foundation, version 4.0.2) for further analysis.

### ELISAs

All assays were performed according to the manufacturers' protocols, unless otherwise specified. The concentrations of ApoA5, ApoC1, ApoC2, ApoC3, ANGPTL3, and ANGPTL8 were measured using the following commercial kits: Human Apolipoprotein A-V sandwich ELISA (LS-F23424; LifeSpan Bioscience, Inc; 1:100 dilution), Human Apolipoprotein CI ELISA Kit (ab108808; Abcam, UK; 1:100 dilution), Apolipoprotein CII ELISA Kit (ab168549; Abcam, UK; 1:200 dilution), Human Apolipoprotein CIII ELISA Kit (ab154131; Abcam, UK; 1:4,000 dilution), Angiopoietin-Like Protein 3 Human ELISA (RD191092200R; BioVendor, Czech Republic; 1:5 dilution), and ELISA Kit for Human Betatrophin (Wuhan EIAab Science Co, China, E11644h; 1:4 dilution), respectively. ANGPTL4 was measured using the Human Angiopoietin-like 4 DuoSet ELISA kit (DY3485; R&D Systems; 1:25 dilution) with some modifications to the manufacturer's protocol (35). The capture antibody was incubated on the plate for 4 h at 37°C, instead of overnight at 4°C. Samples were incubated on the plate overnight at 4°C, instead of 2 h at room temperature. The rest of the assay was performed in accordance with the manufacturer's protocol.

### NMR-based metabolomics

NMR-based metabolomics was performed using the Nightingale platform (Nightingale Health Ltd, Finland). The benefit of this platform is that it provides simultaneous high-throughput analysis of over 200 biomarkers in a single plasma sample including measurements of lipoprotein subclasses, their size, and composition (36). In brief, frozen plasma samples were gently thawed overnight and mixed with an equal ratio of 75 mM sodium phosphate buffer (in 80%/20% H_2_O/deuterium oxide, pH 7.4, with 0.08% sodium 3-(trimethylsilyl) propionate-2,2,3,3-d4 and 0.04% sodium azide). Thawed samples were loaded on either a Bruker AVANCE III 500 MHz spectrometer or a Bruker AVANCE III HD 600 MHz spectrometer in order to collect data on the lipoproteins and the low molecular weight metabolites. After the measurements were completed, the samples underwent a multistep lipid extraction procedure and fractions were analyzed on the Bruker AVANCE III HD 600 MHz spectrometer in order to obtain data on lipid composition. All data were then processed by Bruker in an in-house software and transferred to the end user in a ready-to-analyze form (36).

### Statistical analysis

**Fig. 1:**
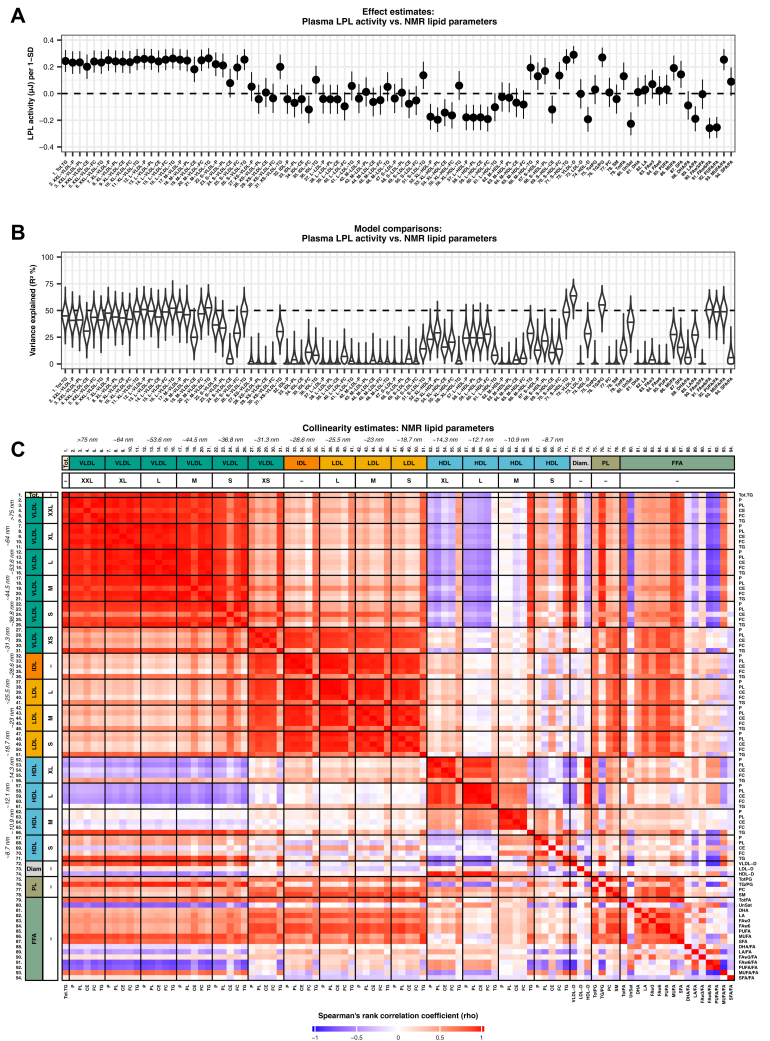
Effects of plasma lipids on LPL activity. A: Forest plot showing the effect of NMR parameters on LPL activity (numbered labels from C) with Bonferroni-corrected 95% CIs. B: Violin plot showing the conditional variance explained (*R*^2^ %) by each NMR parameter. The *x*-axis density curves reflect the frequency distributions that resulted from bootstrapping the *R*^2^ % using 10,000 random resamples. C: Heatmap showing the Spearman's rank correlations between 94 NMR lipid parameters. Complete variable descriptions with abbreviations as well as summary statistics are provided in the supplemental data (supplemental Tables S1 and S2).

Density plots and histograms for the lipid and regulator protein measurements were visually inspected to assess their frequency distributions. The statistical relationships between LPL activity, the regulator proteins, and NMR lipid measurements were evaluated using univariate linear regression and are either reported as the change in LPL activity (expressed in microjoule per second) per 1 SD increase in the continuous *x*-variable (NMR measurements) or per unit of concentration (regulator proteins). To compare the linear regression models against each other, we calculated frequency distributions for their respective conditional variance explained percentage (*R*^2^ %) using bootstrapping with 10,000 random resamples for each model. Correlations between the individual NMR measurements are reported as standardized correlation coefficients (Spearman's rank correlation coefficient). The primary hypothesis was that the plasma concentrations of the known LPL regulators would affect the activity of LPL, as measured by ITC. Thus, to correct for multiple comparisons in our primary analysis, we calculated the *P* value threshold as 0.05/9 ≈ 0.006 using the Bonferroni method. In the NMR data, there was a high degree of multicollinearity between variables (Fig. 1, panel C). Therefore, we used principal component analysis to address the multiple comparison issue (37). The number of hypotheses ultimately corrected for corresponded to the number of principal components that could explain 99.5% of the variance in the data. For our data, hypothesis testing on the NMR parameters corresponded to 22 independent tests (corrected *P* for significance = 0.05/22 ≈ 0.002). All reported confidence intervals and *P* values are corrected for multiple comparisons. Samples with missing values at random were excluded from further analyses. Six samples in the NMR dataset held left-censored missing values (in total 0.4% of all data points), caused by concentrations being below the instrument limit of quantification. To preserve information, these values were imputed by a deterministic minimal value approach using the *imputeLCMD* package. The statistical analyses were performed using R software (free from The R Foundation, version 4.0.2).

## Results

### Activity of LPL in plasma samples as measured by ITC

The physiological parameters of the 117 enrolled patients are presented in Table 1. To directly study the action of LPL on lipids in the plasma samples, we used the novel method based on ITC, which was described previously (27). Before addition of LPL, there was essentially no change in heat production in the sample cell, which was translated into a stable baseline on the thermograms. This illustrated that there are only very low levels of catalytically active endogenous LPL present in human plasma (38). Therefore, lipolysis was studied after addition of a standardized amount of purified bovine LPL. Because of the high reproducibility of this method, and the relatively large volume of sample plasma used per run, each sample was analyzed only once. The three repeated additions of bovine LPL ensured that there was a linear response and thus enough substrate present in the experiment. The baseline change after the first injection was used for further calculations.

**Table 1:** Descriptive statistics for study participants

Measurement	n_obs_	Mean/Median
Age (years)	117	63 (IQR: 53–63; range: 43–63)
Sex	117	
Male	57 (48.7%)	
Female	60 (51.3%)	
Systolic blood pressure (mm Hg)	114	130.5 (SD: ±18.6; range: 89–174)
Diastolic blood pressure (mm Hg)	114	86.7 (SD: ±10.1; range: 65–113)
Body mass index (kg/m^2^)	114	26.1 (IQR: 23.9–29.1; range: 19.0–41.1)
Total cholesterol (mmol/l)	114	5.35 (SD: ±1.10; range: 2.9–8.0)
Total triglycerides (mmol/l)	114	1.35 (SD: ±0.84; range: 0.4–4.9)
LDL cholesterol (mmol/l)	112	3.13 (SD: ±1.00; range: 1.2–5.7)
HDL cholesterol (mmol/l)	113	1.60 (SD: ±0.47; range: 0.7–3.6)
Fasting blood glucose (mmol/l)	114	5.5 (IQR: 5.1–6.0; range: 4.0–8.4)
LPL activity (μJ/s)	117	0.72 (SD: ±0.37; range: 0.05–1.64)
Angiopoietin-like 3 (ng/ml)	117	236 (IQR: 194–301; range: 107–516)
Angiopoietin-like 4 (ng/ml)	116	159 (IQR: 97–493; range: 40–1,489)
Angiopoietin-like 8 (pg/ml)	116	1,397 (IQR: 1,051–1,734; range: 641–4,819)
Apolipoprotein C-I (μg/ml)	116	57 (IQR: 50–66; range: 33–118)
Apolipoprotein C-II (mg/dl)	117	12.0 (IQR: 8.4–21.1; range: 0.9–91.8)
Apolipoprotein C-III (mg/dl)	117	14.0 (IQR: 12.6–17.5; range: 8.2–49.3)
Apolipoprotein A-V (ng/ml)	117	10.8 (IQR: 5.9–19.2; range: 0.9–117.2)

IQR, interquartile range; *n*_obs_, number of complete observations.

Summary statistics are presented as mean with SD and range, or median with IQR and range, depending on the distribution of the data.

As can be seen in Table 1, the LPL activity, as measured by ITC, varied by a factor of 33 between plasma samples from the 117 middle-aged individuals. The lowest recording was 0.05 μJ/s, and the highest recording was 1.64 μJ/s. To explain the large variation in response to the added LPL, we analyzed known protein factors in the plasma samples that might affect the activity of LPL as well as lipoprotein sizes. As determined by the linear regression analysis, one of the strongest determinants of LPL activity was the mean diameters of VLDL and HDL particles (Fig. 1, panels A and B). Thus, their composition was investigated further.

### Effects of physical parameters of plasma lipoprotein particles on LPL activity

The effect of various factors on LPL activity was extensively studied previously (27, 39, 40). It is well known that, although LPL can interact with all major lipoprotein classes, its affinity for the large TRL particles is much higher than for the small cholesterol-rich LDL and HDL (39, 40, 41, 42). It was therefore not surprising that the strongest effector for LPL activity in plasma was the mean diameter of the VLDL particles (Fig. 1, panels A and B). By further analyses we found that the average VLDL diameter was superior to total plasma TG at explaining the ITC-measured LPL activity (Fig. S1). Plasma from overnight fasted individuals is expected to contain low amounts of small TG-poor chylomicrons that are not expected to significantly contribute to the overall lipolysis. The ratio of TG to phospholipids, the VLDL TG levels, and the content of VLDL cholesterol were also determinants of LPL activity (Fig. 1, panels A and B); however, these properties strongly correlated with the size of the VLDL particles (Fig. 1, panel C).

IDL and LDL had no specific effect on the activity of LPL in our assay, with the exception for a few variables that may be attributed to the metabolism of VLDL, such as the TG content of IDL (Fig. 1, panels A and B). One of the strongest inverse correlations with LPL activity in our study was found for the HDL diameter (Fig. 1, panels A and B). The same trend carried over to HDL cholesterol levels and levels of ApoA1 (Fig. 1, panels A and B).

### Effects of plasma lipids on LPL activity

Evaluation of the effect of the total FA composition in plasma on the activity of LPL, as measured by ITC, is complicated (Fig. 1, panels A and B). A limitation of our study is that we only have data for the whole plasma sample, including not only FAs in TG but also FFA, FAs in cholesterol esters, in phospholipids, and in other FA-based lipids. Furthermore, the FA composition of each lipoprotein fraction was not determined. Therefore, one can expect contributions to the lipid composition from lipoproteins that are not substrates for LPL (such as LDL and HDL). LPL has a strong preference for TGs and diglycerides, but it can also hydrolyze phospholipids (43, 44). The contribution of phospholipid hydrolysis to the recorded heat production under our conditions is not known. Taking these caveats into account, there were still significant positive and negative correlations between the LPL activity recorded by ITC and the FA composition of the plasma samples (Fig. 1, panels A and B). The preference of LPL for TG that contain unsaturated FAs is well known from previous studies (45, 46, 47). There was a weak positive correlation between the activity of LPL in plasma, recorded by ITC, and the level of monounsaturated FAs (Fig. 1, panels A and B). The level of saturated FA, and the ratio of saturated FA to total FA, also correlated positively to the recorded LPL activity (Fig. 1, panels A and B). The overall unsaturation level of FAs correlated however negatively with LPL activity (Fig. 1, panels A and B). The ratios of ω-6 to total FA and of PUFA to total FA showed a negative correlation with LPL activity, as did the unspecified unsaturation level of FA and the ratio of linoleic acid to total FA. The negative correlation between LPL activity and the ω-6 FA ratio was one of the strongest negative correlations in our study. However, all these variables were intermediately to highly collinear with the size and lipid content of the VLDL particles (Fig. 1, panel C).

### Effect of known protein regulators of LPL on its activity

**Fig. 2:**
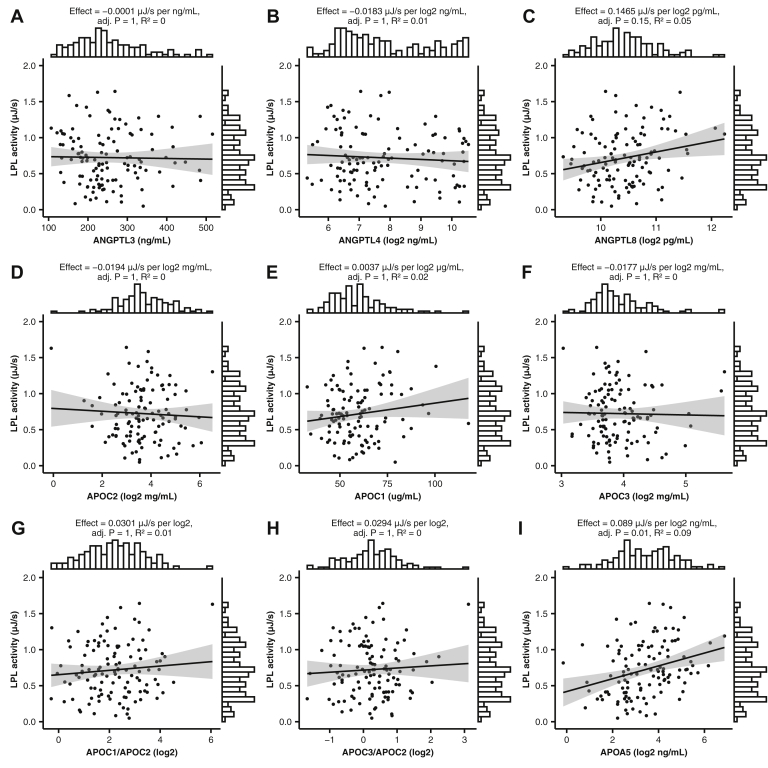
Effect of known protein regulators of LPL activity. A–I: Scattered points with smoothed conditional means (linear regression slopes) and standard error intervals, showing the relationship between LPL activity measured by ITC, and the plasma concentrations of LPL regulator proteins. Effect estimates are presented as LPL activity change (microjoule per second) per 1 SD change in X-variable unit of concentration. The margin histograms show the distributions of the *x* and *y* variables on the opposite side of their respective axes.

We analyzed the levels of the three members of the ANGPTL protein family, which are known to have strong effects on LPL stability, and thereby its activity level (18, 48, 49, 50). The content of ANGPTL3, ANGPTL4, and ANGPTL8, as measured, showed no significant correlation with the activity of LPL when added to the plasma samples (Fig. 2, panels A–C). This could be expected for ANGPTL4 because of the efficient protection of LPL by the presence of TRLs (51), and that most of the ANGPTL4 in plasma is the inactive carboxy-terminal part (18). The lack of effect of ANGPTL3 and ANGPTL8, which were previously shown to work as heterooligomers, may be due to the low levels of ANGPTL8 (17, 23, 24). The blood samples were taken from fasted subjects, and fasting is known to suppress the production of ANGPTL8. Therefore, the measured levels of ANGPTL8 were nearly 100-fold lower than those of ANGPTL3 and ANGPTL4. As a result, it can be expected that there were insufficient amounts of ANGPTL3/8 heterooligomers present in plasma to be able to have an effect on LPL activity.

ApoC1, ApoC2, ApoC3, and ApoA5 were all previously shown to affect LPL activity (12, 15, 52). Of the four Apos, ApoC2 is the most studied and best-known effector acting as a cofactor for LPL. However, the 10-fold difference observed in the levels of ApoC2 in the patients' plasma showed no significant correlation with the recorded LPL activity (Fig. 2, panel D). This supported the previous notion that the normal physiological levels of ApoC2 are sufficient to completely saturate the LPL system (27, 53). Similarly, correlations were neither obtained with the levels of ApoC1 and ApoC3 nor with the ratios between them and ApoC2, and LPL activity (Fig. 2, panels E–H).

ApoA5 levels, however, showed a weak positive correlation with LPL activity (Fig. 2, panel I). Several studies have shown that ApoA5 plays an important role in LPL-mediated lowering of the TG levels in the blood (16, 54, 55). This is quite intriguing in light of that the levels of ApoA5 in plasma are the lowest amongst all the Apos.

The full list of variables, as well as supplementary statistics for the linear regression models, can be found in the supplemental tables (supplemental Tables S1 and S2).

## Discussion

In the current study, our aim was to measure effects of the levels of the known protein regulators of LPL, and of the physical properties of human plasma, on the activity of LPL that was added to the plasma samples. Our results demonstrate that the main determinants for the activity of LPL were the particle sizes and the levels of VLDL and HDL, as well as the concentration of FAs and their degree of saturation in the total plasma lipids. Surprisingly, the physiological variation neither in the levels of ApoC1, ApoC2, or ApoC3 nor the in the levels of ANGPTL3, ANGPTL4, or ANGPTL8 had any significant effect on the recorded LPL activity under the conditions used. The only known regulator of LPL, which showed a weak significant association with activity, was ApoA5 (Fig. 2, panel I).

Our study shows that the concentrations of VLDL and HDL, as measured by the number of particles, were one of the strongest determinants of LPL activity. Interestingly, only the larger fractions of VLDL and HDL contributed (positively and negatively, respectively) to the LPL activity. We have no explanation other than collinearity for the strong positive effects of HDL TG on LPL activity, as HDL contains relatively small amounts of TG. It is likely that HDL TGs are related to VLDL TGs through nonpolar lipid exchange between the lipoprotein particles mediated by cholesteryl ester transfer protein (56). On the other hand, the smallest HDL particles had an opposite effect to the overall effect of HDL. The levels of the smallest most lipid-poor HDLs are known to be increased in CVD, which is opposite to what is known for the total levels of HDL (57).

The effect of the diameter of TRL on LPL activity was expected, as LPL is known to prefer large and less curved lipid particles (39, 40, 41, 42). This may in part be attributed to the binding preferences of its cofactor ApoC2 (40, 58). ApoC2 prefers surfaces or lipoproteins with little curvature, low surface pressure, and low crowding (58, 59, 60). As ApoC2 is distributed between TRL and HDL, the total concentration of ApoC2 in plasma may be a poor indicator of its ability to activate LPL. Previous studies have demonstrated that concentrations of ApoC2 corresponding to about 100 nM result in full activation of LPL (27, 53). In the present study, the median concentration of ApoC2 in the plasma samples was 12.0 μM, with the lowest value of 0.8 μM. Thus, the physiological levels of ApoC2 saturate the LPL machinery and therefore do not directly play a role in the regulation of LPL activity. The absence of significant effects of the levels of ApoC1 and ApoC3 on LPL activity could be explained by the relatively low levels of these proteins in the plasma samples used for ITC. The median level of ApoC1 corresponded to 6.1 μM, and the median level of ApoC3 corresponded to 12.9 μM. Both levels are within the common range reported in the literature. In our previous study, addition of ApoC3 corresponding to 15 μM in human plasma resulted in only a minor decrease in the LPL activity recorded by ITC (27). ApoC1 was previously proposed to be a slightly more potent inhibitor of LPL than ApoC3 (12). In our study, the mean level of ApoC1 was even lower than that of ApoC3 and, consequently, ApoC1 had no observable effect on the recorded LPL activity. Data from our study on plasma samples from fasted individuals are certainly not enough to disprove the potential regulatory role of ApoC1 and ApoC3 on the activity of LPL. The conditions might be different in samples of postprandial plasma that are expected to also contain TG-rich chylomicrons and chylomicron remnants. Such particles may have more surface available for binding of the Apos, and they may therefore have a chance to influence on LPL activity. Furthermore, it is possible that interaction between LPL and GPIHBP1 is necessary for the inhibitory effect of ApoCs on LPL activity to take place (61). Our present results are, nevertheless, in line with previous evidence that the main effect of ApoC1 and ApoC3 does not directly involve inhibition of LPL activity but rather receptor-mediated uptake of TRL remnants by the liver (14, 62, 63). It should be mentioned that most of the observations on the inhibitory effects of ApoC1 and ApoC3 on LPL originate from in vitro studies with concentrations of these proteins that were much higher than that of ApoC2 (12, 64).

The weak positive effect of ApoA5 on the LPL activity recorded by ITC, though completely in line with previous observations in vivo, was somewhat unexpected (16, 54, 55). ApoA5 was previously linked to reduced levels of plasma TG, and mutations in the *APOA5* gene are linked to hypertriglyceridemia (16, 65). However, the stimulatory effect of ApoA5 on LPL activity has been difficult to replicate in vitro. Several factors complicate the understanding of the mechanism of action of ApoA5. First, ApoA5 is found in plasma at very low levels. In our study, the median concentration of ApoA5 was 10.8 ng/ml or 0.3 nM. This concentration corresponds to a single molecule of ApoA5 on one out of 275 VLDL particles. This ratio is far too low for ApoA5 to have a direct effect on LPL, unless ApoA5 is rapidly moving between the VLDL particles. The second aspect, which makes direct interaction of ApoA5 with LPL unlikely, is that both proteins contain regions with a strong positive charge (15, 16). ApoA5 can use the strong positive charge to interact with GPIHBP1 and/or with HSPG. It was proposed, based on this, that ApoA5 could strengthen the bridge between the TRLs and the capillary endothelium. LPL is bound to the same structures and could somehow take advantage of the support by ApoA5 (15, 66). Our ITC measurements do not include this important aspect, because they are conducted with LPL in solution in the absence of any membrane-bound GPIHBP1 and HSPG. Third, the ApoA5 levels were positively correlated to the TG levels (supplemental Table S3). Therefore, we cannot rule out the possibility that the observed positive effect of ApoA5 on LPL activity was due to the increased TG levels, rather than a direct effect of ApoA5 on LPL.

The lack of a direct effect of the levels of the ANGPTLs on the activity of LPL could be explained by results from a previous study that demonstrated that LPL is stabilized and protected from the action of ANGPTL proteins when the enzyme is bound to TRLs (51). Furthermore, in vivo, most of the active LPLs in plasma are found in complex with GPIHBP1, which was previously shown to protect LPL from both spontaneous and ANGPTL-mediated inactivation (17, 34, 67). We cannot exclude that low levels of soluble GPIHBP1 could contribute to the stabilization of the added LPL (68). Another concern is that our experiments were carried out at 25°C, while the body temperature is 37°C. The stability of LPL both in the presence and absence of ANGPTLs is highly dependent on the temperature (69). All factors considered, including our current data, as well as our previous experiments with ITC, suggest that the levels of ANGPTL proteins are not high enough to act on LPL in the plasma compartment at 25°C (27). The levels of ANGPTL8 were extremely low, nearly 100-fold lower than those of ANGPTL3 and ANGPTL4. This was due to the fact that the plasma samples were obtained from fasted individuals and/or possibly to suboptimal quantification by the immunoassay used because of complex formation between ANGPTL8 and ANGPTL3 or ANGPTL8 (18). Therefore, effects of ANGPTL8, and of its partner, ANGPTL3, were expected to be marginal in our study.

Finally, levels of plasma FAs and their saturation showed significant correlations with the recorded LPL activity in our analyses. Interpretation of these findings is complicated by the fact that the FAs originated from not only TG but also other lipid substrates and from lipids that are not hydrolyzed by LPL including FFA bound to albumin. FFAs are known to inhibit LPL activity by several mechanisms resulting in strong product inhibition (70). This was not expected to occur during our ITC recordings, as human plasma contains high amounts of albumin, and the measurements were performed under conditions when the hydrolysis reaction was linear with time and with the amount of added enzyme. Even though LPL prefers TG as substrate, some phospholipids will also be hydrolyzed. The hydrolysis rate for phospholipids is slower than that for TG (43, 44), and they may therefore act as competitive inhibitors. It is expected that differences in the FA composition of the phospholipids could affect their relative rate of hydrolysis by LPL. Another important aspect is that the lengths and degree of saturation of the FAs contained in the lipoprotein lipids is likely to affect the fluidity and other physical properties of the surface layer of the lipoproteins. This may impact on the binding of proteins to the lipoproteins, including Apos and LPL. It may also influence on the fraction of TG that can be present in the surface layer of the lipoproteins (71). The two-dimensional concentration of TG in the surface layer is probably one of the most important determinants for the rate of hydrolysis by LPL. The catalytic reaction must occur at, or close to, the surface where there is access to water.

In conclusion, our results show that the physiological levels of the known protein regulators of LPL in human plasma are not high enough to influence the rate of lipolysis by added LPL. The levels of the ANGPTL proteins had no significant effect on the recorded LPL activity as measured at 25°C. This may be because ANGPTL-mediated inhibition of LPL activity is highly temperature dependent and presumably stronger at 37°C; or that the ANGPTL8 levels were very low because of fasting; or that in the body, the ANGPTL proteins either act in the subendothelial space, as previously suggested for ANGPTL4, or that inactivation is dependent on that LPL is attached to the endothelium via GPIHBP1 and/or HSPG. Such conditions could not be replicated in our ITC setup. We demonstrate that, despite the fact that ApoC1 and ApoC3 inhibit LPL activity in vitro, their physiological levels in human plasma are too low to inhibit the activity of added LPL. Similarly, the variation in the plasma levels of ApoC2 did not significantly influence on the LPL activity recorded during the ITC experiments. This supports the previous view that ApoC2 is normally present at concentrations that are sufficient to saturate LPL. An interesting possibility is, however, that the distribution of ApoC2 between TRL and HDL could be an important determinant for the rate of lipolysis. Our study identified a plasma lipid signature, including both FFA and lipoprotein parameters associated with the mean VLDL particle size, that strongly influences the rate of lipid hydrolysis by LPL in plasma.

## Data availability

The raw data used for the analyses in this study are stored on a local off-line server. Data can be made available upon reasonable request with regard to local data security legislation (requests: fredrik.landfors@umu.se). The R code used in this study is available in an online code repository (www.github.com/fredlandfors/VIPVIZA-LPL).

## Supplemental data

This article contains supplemental data.

## Conflict of interest

G. O. is a board member and shareholder in Lipigon Pharmaceuticals AB. All other authors declare that they have no conflicts of interest with the contents of this article.

## Supplemental data

Supplemental Figure S1

Supplemental Figures and Table captions
