# Supplementary figures and images for: Lipoprotein size is a main determinant for the rate of hydrolysis by exogenous LPL in human plasma
Source: J Lipid Res. 2021 Oct 26;63(1):100144. doi: 10.1016/j.jlr.2021.100144 (PMC8953621; doi:10.1016/j.jlr.2021.100144)

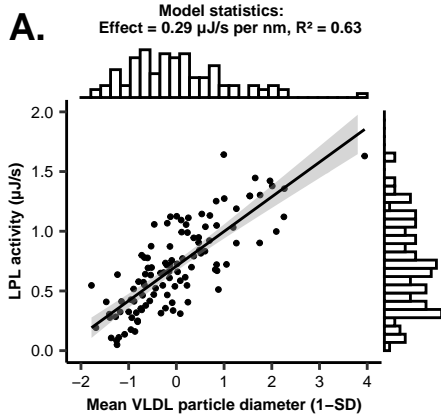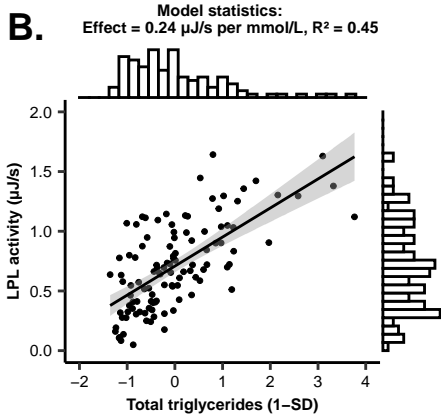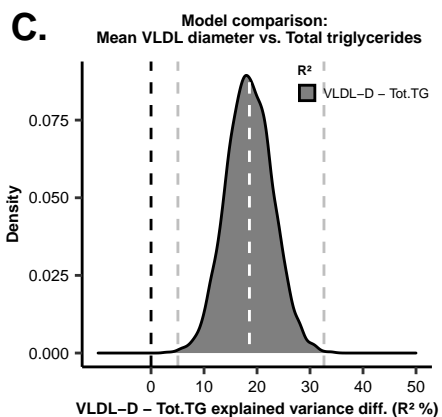

Supplement: Supplemental Figure S1 [file mmc2.pdf]
