# Supplementary material for: Lipoprotein size is a main determinant for the rate of hydrolysis by exogenous LPL in human plasma
Source: J Lipid Res. 2021 Oct 26;63(1):100144. doi: 10.1016/j.jlr.2021.100144 (PMC8953621; doi:10.1016/j.jlr.2021.100144)
Supplement: Supplemental Figures and Table captions [file mmc3.docx]

**SUPPLEMENTARY TABLE 1.** Variable descriptions and summary statistics for 94 NMR lipid measurements shown in Figure 1, panels A-C.

**SUPPLEMENTARY TABLE 2.** Model statistics for the linear regression models shown in Figure 1, panels A-B.

**SUPPLEMENTARY TABLE 3.** Model statistics for the linear regression models of regulator proteins on NMR lipid measurements.

**FIGURE S1. The average VLDL diameter is superior to total plasma triglycerides at explaining ITC-measured lipoprotein lipase activity.** The LPL activity conditional variance explained (R^2^) was compared using two models: **(A)** when using the mean VLDL diameter variable as the independent variable or **(B)** when using the total triglyceride levels as the independent variable**. (C)** We find that the VLDL-D model explains 18.6 percentage points more of the LPL activity measurement variance when compared to the Tot.TG model. In addition, we bootstrap the model differences using 10 000 random resamples and find that this improvement is statistically significant (Bonferroni-corrected 95 % CI: 5.1 – 32.3 %).
